# Right Atrium Tumor Extension Through the Inferior Vena Cava. Considerations About Nine Cases Operated Under Cardiopulmonary Bypass

**DOI:** 10.21470/1678-9741-2019-0053

**Published:** 2019

**Authors:** Fernando Chaud, Silvio Tucci Junior, Solange Bassetto, Rodolfo Borges dos Reis, Alfredo José Rodrigues, Walter Vilella de Andrade Vicente, Paulo Roberto Barbosa Evora

**Affiliations:** 1 Department of Surgery and Anatomy, Faculdade de Medicina de Ribeirão Preto da Universidade de São Paulo (FMRP-USP), Ribeirão Preto, SP, Brazil.; 2 Department of Pathology, Faculdade de Medicina de Ribeirão Preto da Universidade de São Paulo (FMRP-USP), Ribeirão Preto, SP, Brazil.

**Keywords:** Adrenocortical Carcinoma, Carcinoma, Renal Cell, Vena Cava, Inferior, Circulatory Arrest, Deep Hypothermia, Kidney, Urological Surgical Procedures, Treatment Outcome

## Abstract

**Introduction:**

Adrenocortical and renal cell carcinomas rarely invade the right atrium (RA). These neoplasms need surgical treatment, are very aggressive and have poor prognostic and surgical outcomes.

**Case series:**

We present a retrospective cohort of nine cases of RA invasion through the inferior vena cava (four adrenocortical carcinomas and five renal cell carcinomas). Over 13 years (2002-2014), nine patients were operated in collaboration with the team of urologists. Surgery was possible in all patients with different degrees of technical difficulty. All patients were operated considering the imaging examinations with the aid of CPB. In all reported cases (renal or suprarenal), the decision to use CPB with deep hypothermic circulatory arrest (DHCA) on surgical strategy was decided by the team of urological and cardiac surgeons.

**Conclusion:**

Data retrospectively collected from patients of public hospitals reaffirm: 1) Low incidence with small published series; 2) The selected cases did not represent the whole historical casuistry of the hospital, since they were selected after the adoption of electronic documentation; 3) Demographic data and references reported in the literature were presented as tables to avoid wordiness; 4) The series highlights the propensity to invade the venous system; 5) Possible surgical treatment with the aid of CPB in collaboration with the urology team; 6) CPB with DHCA is a safe and reliable option; 7) Poor prognosis with disappointing late results, even considering the adverse effects of CPB on cancer prognosis are expected but not confirmed.

**Table:** 

Abbreviations, acronyms & symbols	
ACC	= Adrenocortical carcinoma
CPB	= Cardiopulmonary bypass
CT	= Computed tomography
DHCA	= Deep hypothermic circulatory arrest
IVC	= Inferior vena cava
MRI	= Magnetic resonance imaging
PET	= Positron emission tomography
PTFE	= Polytetrafluoroethylene
RA	= Right atrium
RCC	= Renal cell carcinoma

## INTRODUCTION

Adrenocortical carcinoma (ACC) and renal cell carcinoma (RCC) are aware malignancies that occasionally present extending into the right atrium (RA) through the inferior vena cava. Patients can present with a variety of signs and symptoms, depending on the extent of the tumor. These neoplasms demand surgical treatment, are very aggressive and have poor prognosis and surgical outcomes. Therefore, this unusual pathological situation has to be in mind of the “heart team”. The discovery of a mass in the right atrium obliges the clinician to perform a broad differential diagnosis between a primary cardiac tumor (myxoma being the most frequent), invasion of an extracardiac tumor, vegetations on the tricuspid valve and atrial thrombus. Tumor extension with vena cava thrombosis is a relatively frequent complication of renal carcinoma, but only exceptionally reaches the right atrium. It is also exceptional that this was a chance finding in an asymptomatic patient^[1]^.

As an overview, Castro-Dominguez et al.^[2]^ stated that ACC is a highly aggressive malignant neoplasm with an incidence rate of 1 to 2 cases per million people per year. Overall 5-year survival is poor, ranging from 15 to 44% in reported series. Multimodality imaging with echocardiogram, computed tomography (CT), positron emission tomography (PET) and magnetic resonance imaging (MRI) aids not only in establishing the diagnosis but also in anatomic evaluation to determine the best surgical approach^[2]^.

According to Locali et al., based on a series of 14 cases, these tumors are routine in urological surgery. But they are important in the context of cardiovascular surgery due to possible complications with intracaval and/or intracardiac thrombi. Studies in this area, however, are mostly case reports or case series with small sample numbers. Due mainly to the rarity of this complication, few studies have been performed with larger case numbers, providing reliable conclusions^[3]^.

Therefore, the objective of this presentation was based on the relative scarcity of reported cases, presenting nine cases of RA invasion through the inferior vena cava (four adrenocortical and five renal tumors) performed over 13 years.

### Cases Series

Over 13 years (2002-2014), nine patients were operated in collaboration with the team of urologists. The patients were allocated into 2 groups presented in Table 1.

**Table 1:** Demographic data.

	Register	Name	Age (years old)	Gender	CPB time (min)	Date of surgery	Survival time (months)	Diagnosis
Suprarenal gland group	0874011J	L.F.B	52	Male	165	Oct 15, 2008	PO	Malignant tumor of suprarenal gland
	0591325D	A.O.S	03	Male	75	Jan 25, 2002	6	Malignant tumor of suprarenal gland
	0713472A	C.G.B	05	Female	85	Nov 12, 2004	15	Malignant tumor of supra renal gland
	1091646A	R.R.V	62	Male	195	March 20, 2012	PO	Malignant tumor of supra renal gland
Renal group	0583349A	M.J.M	89	Male	215	May 22, 2002	PO	Malignant tumor of kidney exceptrenal pelvis
	0836581E	N.A.F.O	61	Female	60	Dec 20, 2007	143	Malignant tumor of kidney except renal pelvis
	1074537D	P.C.P	60	Male	140	Dec 08, 2011	PO	Malignant tumor of kidney except renal pelvis
	1097989J	D.F.V	57	Female	165	March 15, 2012	12	Malignant tumor of kidney except renal pelvis
	1235671J	B.A.B	55	Male	175	May 9, 2014	12	Malignant tumor of kidney except renal pelvis

**Fig. 1:**
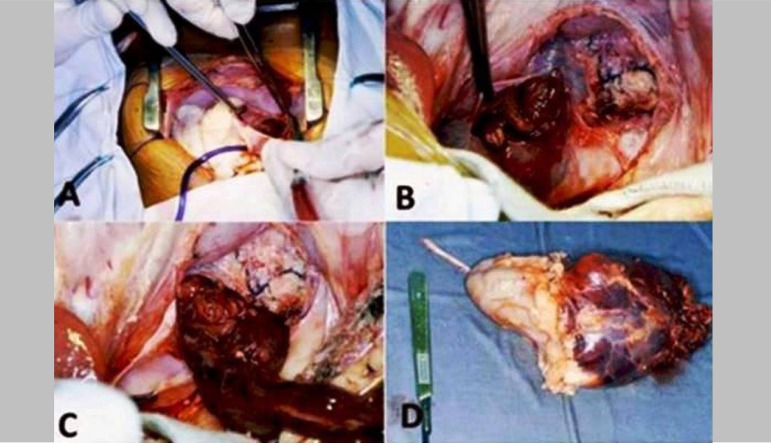
Intraoperative pictures (patient on CPB). A=Right atrium opening; B=Tumor protruding into the right atrium; C=Intracardiac tumor excision; D=Macroscopic aspect of the excised tumor

**Fig. 2:**
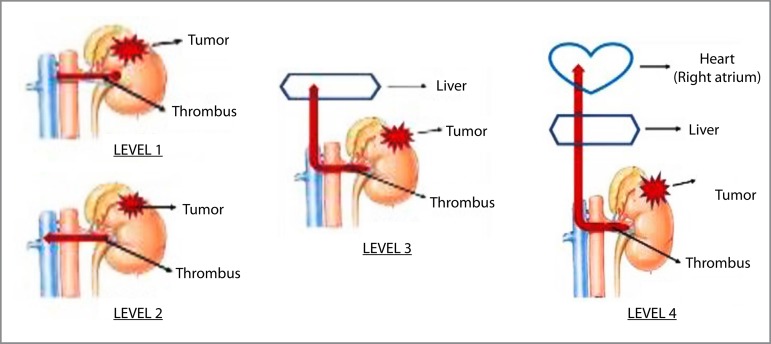
Classification of tumor thrombus level according to the Mayo staging system. (Adapted from Calero A, Armstrong PA. Semin Vasc Surg. 2013 Dec;26:219-25).

Suprarenal group (n=4): Gender (3 males, 1 female); Age (03, 05, 52, 62 years old, respectively); CPB time (165, 75, 85, 195 min, respectively); mortality rate (two patients died in the immediate postoperative day; two survived 6 and 15 months, respectively). Renal group (n=5): Gender (3 males, 2 females); Age (55, 57, 60, 61, 89 years old, respectively); CPB time (175, 165, 140, 60, 215 min, respectively); mortality rate (two patients died in the immediate postoperative day; two survived 12 months, and one survived 143 months, respectively). The renal group did not consider the renal pelvis. Figure 1 shows an intraoperative renal carcinoma with invasion of the inferior vena cava. The CPB data are presented in Table 1. All cases submitted are classified as Level 4 according to the Mayo Clinic classification (Figure 2)^[4]^.

**Fig. 3:**
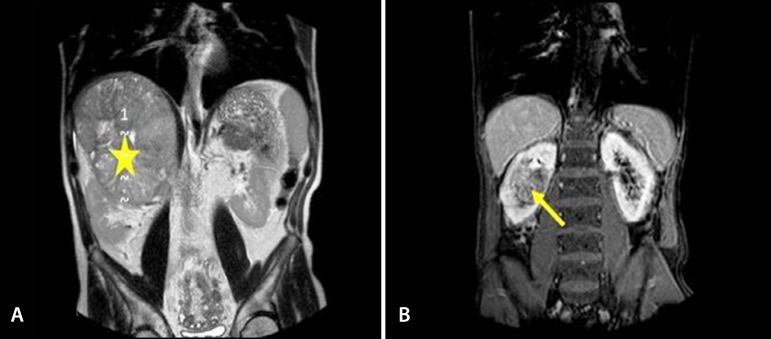
CT image. A=suprarenal tumor (*); B=renal tumor (arrow).

Patients were operated on mainly considering imaging CT scans that were good enough for tumor observations (Figure 3).

In all reported cases (renal or suprarenal), the use of CPB with deep hypothermic circulatory arrest (DHCA) and the surgical strategy were decided by the team of urological and cardiac surgeons. In summary, through a modified chevron incision, starting two fingerbreadths below the right costal margin and extending laterally to the midaxillary line, the kidney was exposed and mobilized laterally and posteriorly, the perirenal collateral circulation and the renal artery were ligated. Infrarenal inferior vena cava (IVC) and the contralateral renal vein were dissected. Liver mobilization, when necessary, was performed. After the abdominal step, a median sternotomy was performed, the pericardium was opened, CPB was installed and DHCA was carried out (Figure 1).

**Fig. 4:**
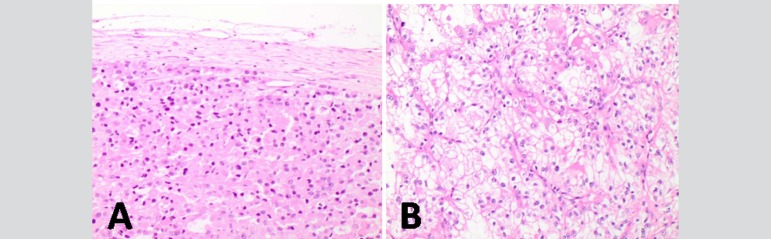
Histology: A=Sample of adrenocortical carcinoma attached to the venous wall (H&E, 200X); B=Clear cell renal cell carcinoma with scant stroma and delicate vasculature (H&E, 400X).

Only once the aorta was clamped for infusion of cardioplegia in a patient who had a mild coronary lesion. In other cases, induced ventricular fibrillation was expected, always maintaining good drainage of the left chambers. After CPB, the IVC was incised from the liver border to the renal vein and the tumor was removed in block. After tumor removal, saline was injected to wash the proximal region of the IVC, low flow CPB was carried out, and body rewarming according to the total circulatory arrest protocol. When necessary, polytetrafluoroethylene (PTFE) or bovine pericardium patches were used for the IVC reconstruction. Histological samples are presented in Figure 4, confirming the diagnosis.

## DISCUSSION

As already mentioned, adrenocortical carcinomas and renal cell carcinomas (RCC) are rare malignancies. According to Spanish data, collected between early 1975 and April 1997, among 212 patients who underwent surgery for RCC, only 2 cases showed right atrial extension^[5]^. Among the metastatic tumors of the heart, those arising from the genitourinary system are amongst the most common^[6]^. Most of the case reports references were presented in Table 2.

**Table 2:** Selected references.

Castro-Dominguez et al.	Pak J Med Sci. 2017 Mar-Apr; 33(2):510-512	1 case (male)
Lau et al.	Ann Thorac Surg. 2016 Sep;102(3):836-842	1 case
Levin et al.	Radiol Case Rep. 2016 Feb 17;10(2):1084.	1 case
Naffaa et al.	BMJ Case Rep. 2014 Mar 18;2014. pii: bcr2014203794. doi: 10.1136/bcr-2014-203794.	1 case
Patil et al.	BMJ Case Rep. 2013 Oct 14;2013. pii: bcr2013200804. doi: 10.1136/bcr-2013-200804.	1 case
Kumar et al.	J Clin Imaging Sci. 2013 Aug 31;3:32. doi: 10.4103/2156-7514.116186. eCollection 2013.	(1 case female)
Swan et al.	Ann Surg Oncol. 2012 Apr;19(4):1275. doi: 10.1245/s10434-011-2203-4. Epub 2012 Jan 26.	(1 case female)
Senthil et al.	Jpn J Radiol. 2012 Apr;30(3):281-3. doi: 10.1007/s11604-011-0037-4. Epub 2011 Dec 17.	(1 case)
Wright et al.	J Cardiovasc Surg (Torino). 2008 Feb;49(1):79-81.	(1 case)
Yeh et al.	Am J Surg. 2006 Aug;192(2):209-10. Virilizing adrenocortical carcinoma with cavoatrial extension.	(1 case)
Ochi et al.	Int J Urol. 2006 Mar;13(3):202-5.	(1 case) 4 renal cases
Nagasaki et al.	Clin Pediatr Endocrinol. 2004;13(1):25-32. doi: 10.1297/cpe.13.25. Epub 2004 Jul 7.	(1 case)
Hisham et al.	Asian J Surg. 2003 Jan;26(1):40-2.	(1 case)
Hoang et al.	Mod Pathol. 2002 Sep;15(9):973-8.	(1 case)
Chesson et al.	Scand J Urol Nephrol. 2002 Feb;36(1):71-3.	(1 case)
Peix et al.	Ann Chir. 1998;52(4):357-63.	(1 case)
Chiche et al.	Surgery. 2006 Jan;139(1):15-27.	(4 cases)
Rosen et al.	Cardiovasc Ultrasound. 2003 May 16;1:5.	(1 case)
Lee et al.	J Am Soc Echocardiogr. 1998 Jan;11(1):86-8.	(1 case)
Hedican et al.	J Urol. 1997 Dec;158(6):2056-61.	(15 cases)
Godine et al.	Pediatr Radiol. 1990;20(3):166-8; discussion 169.	(3 cases)
Ohnishi et al	J Cardiol. 1990;20(2):377-84.	(1 case)
Cheung et al.	Cancer. 1989 Aug 15;64(4):812-5.	(1 case)

Patients can present with a variety of signs and symptoms, depending on the extent of the tumor. CT scan of chest and abdomen represents the gold standard in ACC staging, while magnetic resonance imaging (MRI) is preferred for tumor thrombus characterization. Complete surgical resection is the only curative option for localized disease. Kidney-sparing surgery should be performed when possible. Hedican and Marshall^[7]^ reported three patients with adrenocortical carcinoma with tumor thrombus and reviewed 26 patients described in the literature from 1972 to 1997 regarding presentation, management and outcome. Among 15 patients, Nakahoma et al.^[8]^ presented a case that seems to be the 8^th^ case report of left adrenocortical cancer with tumor thrombus extension into IVC and right atrium. More recently, Castro-Dominguez et al.^[2]^ reported one case of a large ACC with extension to the IVC and right atrium (RA). Our nine cases will be added to the medical literature without changing the low incidence paradigm with small published series, because they do not represent the whole historical casuistry of the hospital, since they are chosen after the adoption of electronic documentation.

DHCA is the most commonly used method and allows complete tumor resection without increasing operative risk. The cardiothoracic team, considering the unusual situation, opted for routine cardiac surgeries (median sternotomy, careful venae cavaes dissections). Deep hypothermia was employed, and due to technical difficulty in one patient, the heart was arrested with cold blood potassium cardioplegia. However, further studies are needed to evaluate the possible role of alternative methods compared to deep hypothermic circulatory arrest^[9,10]^.

The outcome evaluation confirmed the well-established poor prognosis with disappointing results (two patients survived for more than 12 years, four died in the early postoperative period and, among the others, four patients did not live longer than 12 months). One patient died during surgery due to intractable blood clotting disorder; two developed vasospastic syndrome and died on the second postoperative day; a third patient died during the performance of the arteriovenous fistula to treat severe acute sufficiency. The other five patients presented dissemination of the disease and died due to respiratory failure.

About the possible influence of the type of neoplasia, we did not find any publications directly correlating the type of neoplasia with possible dissemination caused or favored by CPB. In other words, it cannot be said that a particular kind of cancer is more susceptible to propagation by CPB. The application of CPB in oncologic patients is still controversial, with the possible disadvantages of hematogenous dissemination of tumor cells. There are two possible mechanisms through which CPB might contribute to the hematogenous dissemination of tumor cells. First, tumor cells contaminated in the blood reservoir might spread through the arterial cannula. Second, the CPB homeostasis imbalance may contribute to the dissemination of neoplastic cells preoperatively suppressed by the host defense system. Finally, further research is needed to know whether the transient immunosuppression associated with CPB can promote the spread and growth of pre-existing cancer cells. However, adverse effects of CPB on cancer prognosis are expected but have not been confirmed^[11,12]^.

## CONCLUSION

The present data retrospectively collected from public hospital patients reaffirm: 1) Low incidence with small published series; 2) The selected cases did not represent the whole historical casuistry of the hospital, since they are selected after the adoption of electronic documentation; 3) Demographic data and references reported in the literature were presented as tables to avoid wordiness; 4) The series highlights the propensity to invade the venous system; 5) Possible surgical treatment with the aid of CPB in collaboration with the urology team; 6) CPB with DHCA is a safe and reliable option; 7) Poor prognosis with disappointing late results, even considering that adverse effects of CPB on cancer prognosis are expected but have not been confirmed.

**Table:** 

Author's roles & responsibilities	
FC	Acquisition, analysis, or interpretation of data for the work; final approval of the version to be published
STJ	Acquisition, analysis, or interpretation of data for the work; final approval of the version to be published
SB	Acquisition, analysis, or interpretation of data for the work; final approval of the version to be published
RBR	Acquisition, analysis, or interpretation of data for the work; final approval of the version to be published
AJR	Substantial contributions to the conception or design of the work; final approval of the version to be published
WVAV	Substantial contributions to the conception or design of the work; final approval of the version to be published
PRBE	Substantial contributions to the conception or design of the work; or the acquisition, analysis, or interpretation of data for the work; drafting the work or revising it critically for important intellectual contente; final approval of the version to be published
